# Stepped care for depression and anxiety: from primary care to specialized mental health care: a randomised controlled trial testing the effectiveness of a stepped care program among primary care patients with mood or anxiety disorders

**DOI:** 10.1186/1472-6963-9-90

**Published:** 2009-06-05

**Authors:** Wike Seekles, Annemieke van Straten, Aartjan Beekman, Harm van Marwijk, Pim Cuijpers

**Affiliations:** 1 Department of Clinical Psychology, VU University, Amsterdam, The Netherlands; 2 Department of Psychiatry, VU University Medical Centre, Amsterdam, The Netherlands; 3 Department of General Practice, VU University Medical Centre, Amsterdam, The Netherlands; 4 EMGO institute, VU University Medical Centre, Amsterdam, The Netherlands

## Abstract

**Background:**

Mood and anxiety disorders are highly prevalent and have a large impact on the lives of the affected individuals. Therefore, optimal treatment of these disorders is highly important. In this study we will examine the effectiveness of a stepped care program for primary care patients with mood and anxiety disorders. A stepped care program is characterized by different treatment steps that are arranged in order of increasing intensity.

**Methods:**

This study is a randomised controlled trial with two conditions: stepped care and care as usual, whereby the latter forms the control group. The stepped care program consists of four evidence based interventions: (1) Watchful waiting, (2) Guided self-help, (3) Problem Solving Treatment and (4) Medication and/or specialized mental health care. The study population consists of primary care attendees aged 18–65 years. Screeners are sent to all patients of the participating general practitioners. Individuals with a Diagnostic and Statistical Manual of mental disorders (DSM) diagnosis of major depression, dysthymia, panic disorder (with or without agoraphobia), generalized anxiety disorder, or social phobia are included as well as individuals with minor depression and anxiety disorders. Primary focus is the reduction of depressive and anxiety symptoms. Both conditions are monitored at 8, 16 and 24 weeks.

**Discussion:**

This study evaluates the effectiveness of a stepped care program for patients with depressive and anxiety disorder. If effective, a stepped care program can form a worthwhile alternative for care as usual. Strengths and limitations of this study are discussed.

**Trial Registration:**

Current Controlled Trails: ISRCTN17831610.

## Background

Depressive and anxiety disorders are highly prevalent [1]. Currently, depressive disorders are the fourth disorder worldwide in terms of disease burden, and will be the disorder with the highest disease burden in high-income countries by 2030 [2]. Anxiety and depression can cause serious functional impairment, reduced quality of life [3,4], high levels of public service usage and huge economic costs [5,6]. Comorbidity is high among psychiatric disorders in general [1]. Almost half of the people who have ever suffered from a psychiatric disorder have had more than one. Comorbid anxiety is the rule rather than the exception with up to 60% of the patients with major depressive disorder also suffering from an anxiety disorder [7]. Because of this high comorbidity and the difficulties in distinguishing the two disorders without extensive diagnostic interviews, this study includes individuals with depressive disorders as well as with anxiety disorders.

There are ongoing discussions regarding the effectiveness of the management of common mental health problems in general practice settings. Both anxiety disorders and depression can be treated effectively by pharmacotherapy as well as psychotherapy [8-15]. Although evidence based clinical guidelines are available, initiation and adherence to effective treatment are usually poor [16-19]. Mood and anxiety disorders often remain unrecognised and therefore untreated in primary care [20]. Furthermore, it has been demonstrated that even when depression has accurately been recognized, still only few patients receive adequate care [19]. According to several studies [21-23] many patients receive antidepressants immediately after the diagnosis was determined, however, only few patients receive adequate dosage and duration of (antidepressant) medication. Approximately 30% of depressed primary care patients stop using antidepressant medications within the first month of treatment, while only 40% reach the recommended therapeutic dosage of antidepressant medication [24]. It is also important to note that the majority of primary care patients prefer psychotherapy as a treatment [25]. Clearly, the gap between research findings and clinical practice is wide in the management of depression in primary care [26].

Through its objective of initiating interventions at the right time and as adequately as possible, the stepped care model could provide a solution for the problem of applying effective, evidence based care for depression and anxiety. Care is offered not earlier or more intense than necessary and not later or less intense than needed [27,28]. In a stepped care model, all patients start with an evidence-based treatment of low intensity as a first step. Progress is monitored and patients, who do not respond adequately can 'step up' to a subsequent treatment of higher intensity [29]. An important feature of the stepped care model is that the model is self-correcting. Self-correcting means that the results of treatments and decisions about treatment provisions are monitored systematically and where necessary changes are made ('stepping up') if current treatments are not achieving significant health improvement [27]. A care manager coordinates a stepped care program, preferably a paraprofessional who supports the primary care clinic handling psychiatric problems. In The Netherlands the most likely candidate for this role is a psychiatric nurse. This care manager coordinates the monitoring of the patients, provides the first treatments in the stepped care model and refers the patient to the appropriate mental health care specialist if necessary.

Although the stepped care model seems to be a promising care model [27-30] to increase the effectiveness and efficiency of the mental health system, its effectiveness compared with care as usual (CAU) has not been studied yet [27-29]. We will describe the protocol of a study on the (clinical) effectiveness of this program versus care as usual with patients who are diagnosed with a minor or major mood and/or anxiety disorder.

## Methods

### Study design

This study is a randomised controlled trial. We recruit patients by screening all patients of the participating GPs during an inclusion period of 1,5 years. All patients with a positive screen for depression and/or anxiety are screened again after 4 to 6 weeks. Those with a second positive screening (baseline) are contacted by telephone for a diagnostic interview. During this interview all in- and exclusion criteria are checked. Those who are eligible for participation are being asked for informed consent to participate in the study. All patients who give informed consent are subsequently randomised to either (1) stepped care or (2) care as usual. All patients are asked to fill out questionnaires again after 8, 16 and 24 weeks. The study protocol, information brochure and informed consent are approved by the Medical Ethics Committee of the VU University Medical Center (registration number 2006/248).

### Inclusion and exclusion criteria

We include adults aged 18 – 65 years with one or more of the following DSM-IV diagnoses: major depression (single episode or recurrent), dysthymia, panic disorder (with or without agoraphobia), social phobia or generalized anxiety disorder, including comorbid diagnoses. We also include patients with a minor depression or a minor anxiety disorder. We use the DSM-IV research criteria to define minor depression. The main difference between the criteria of minor and major depression is that for minor depression only two to four out of nine symptoms have to be present, of which at least one has to be a core symptom (depressed mood or markedly diminished interest or pleasure in all, or almost all, activities). As there are no DSM criteria, we define a minor anxiety disorder as a score of 12 or more on the Hospital Anxiety and Depression Scale [31] and dysfunctioning in daily life (household, work and/or social relations). Exclusion criteria are psychotic or bipolar disorder, current (< 2 months) treatment (medical/psychotherapy) for psychological problems, prominent suicide ideation, severe alcohol problems (> 20 on the Alcohol Use Disorders Identification Test (AUDIT)[32], no motivation for treatment and insufficient knowledge of the Dutch language.

### Recruitment

#### Recruitment of GPs

In the present study we collaborate with two mental health centers in Amsterdam (GGZ inGeest and Mentrum). Both of these mental health centers employ psychiatric nurses and psychologists, who work for a few hours per week in a GP practice. Usually, GPs refer patients to these psychiatric nurses/psychologists for short-term treatments. First we want to approach psychiatric nurses and psychologists and secondly we want to invite the corresponding GPs to collaborate in this study.

#### Recruitment of patients

Patients are recruited by screening all patients of the participating GPs during the inclusion period of 1,5 year. All patients with a positive screen for depression and/or anxiety are assigned to a watchful waiting period of 4 weeks. After 4 weeks every patient is screened again to exclude the patients who recover spontaneously. This second screener is the baseline questionnaire (T0) and is sent to each patient together with general information about the project and an informed consent form. Two weeks later the patients are approached for a diagnostic interview (Composite International Diagnostic Interview: CIDI) [33] by telephone to check for in- and exclusion criteria. Patients who meet the inclusion criteria *and *return their informed consent are randomised.

### Randomisation

Randomisation takes place at an individual level. Patients are randomised into two groups, stratifying by care manager and using blocks of 4 to prevent overburdening the care managers. An independent researcher not involved in the current project uses computer generated block randomisation to produce sealed envelops. After every inclusion the researcher opens a sealed envelop.

### Intervention

The stepped care intervention consists of four steps: *(1) Watchful waiting*. Patients with mood and anxiety disorders often recover spontaneously over time [34]. In the first step the patients receive no treatment for four weeks. In this project, only patients who still show symptoms of anxiety/depression after the watchful waiting period, are included. They start with (2) *Guided self-help*. Guided self-help starts with one 30 minute session with a care manager. This session enables the care manager to check exclusion criteria (e.g., severe psychopathology), to give psychoeducation (e.g., advice on lifestyle) and to explain the self-help interventions. In this study we have two different self-help interventions available. The first is a generic intervention based on problem solving treatment, which aims at patients with symptoms of mood and/or anxiety disorders. The Dutch version of self-examination therapy is called "*Alles onder controle*" and is available as a book and through the Internet. The patient can choose to get feedback by email or by telephone. In this study feedback is provided by coaches (members from the research group) from the VU University. The focus of the feedback is to guide the patient through the intervention by motivating and activating them. When patients send their assignments to their coach, they receive feedback within 3 working days. The second self-help intervention is specifically aimed at patients with phobias and is based on exposure therapy. In this course patients first have to make a list of all the situations that provoke anxiety and put them in order of intensity. Next they have to make a plan to practice exposure to these situations based on this anxiety hierarchy. This course takes six weeks to complete and is only available as a book. Feedback is therefore provided by telephone. During the first session, the care manager and the patient together decide which self-help course is most suitable. Patients who do not recover using self-help treatment start with (3) *Problem Solving Treatment (PST)*. PST is a short psychological intervention, 5 sessions of 45 minutes each, provided by the care manager [35]. The treatment protocol is based on '*Problem-solving treatment for anxiety and depression: A practical Guide*' by Laurence Mynors-Wallis [36]. Recent studies show that PST is an effective treatment for major depression as well as for more general, emotional disorders containing depressive and anxiety symptoms [35,37-42]. Training in PST was given by a problem solving treatment expert in a two-day course followed by weekly group supervision sessions. Patients who are unresponsive to this treatment will proceed to the last step of the stepped care program (4) *Pharmacotherapy and/or specialized mental health care*. In case patients do not recover from PST they have one more session with the care manager to discuss the next step: either pharmacotherapy or more specialized mental health care. In case a patient chooses pharmacotherapy, the care manager sets up an appointment for the patient with the GP. In case the patient chooses specialized mental health care, the care manager sets up an appointment with a mental health care specialist.

#### Exceptions

Even though there is no clear evidence that patients with more severe symptoms of anxiety or depression do not benefit from low intensity (self-help) interventions, we decided that patients with more severe disorders should be referred to more specialized mental health care and/or pharmacotherapy directly and skip the preceding steps. Severity of the disorders is based on questions about daily functioning on the Work and Social Adjustment Scale (WSAS) [43]. If the patient experiences extreme dysfunctioning (score of 8 or higher) on minimal three of the four domains (household, work, social relations and social activities) he will be directed immediately to the fourth step of the stepped care program.

#### Care as Usual

Patients randomised to the 'care as usual' will be informed about this outcome. They are informed that they can choose to find (mental) health care and are allowed to discuss this with their GP.

#### Assessments and definition of recovery

After each step in the stepped care intervention, patients are monitored: after 8 weeks (T1), 16 weeks (T2) and 24 weeks (T3). During each assessment we measure depressive symptoms, symptoms of anxiety and daily functioning. Recovery is defined as a score below the cut-off score on all three of the following questionnaires: having a score of less than 14 on the Inventory of Depressive Symptomatology (IDS) [44], a score of less than 8 on the Hospital Anxiety and Depression Scale [31] and a score of less than 6 on the WSAS [43]. This definition of recovery is based on several studies [44-48]. On account of the outcome of the monitor, we decide whether the patient is recovered or is 'stepping up' to the next step in the stepped care model.

### Instruments

#### Screening

##### Kessler Psychological Distress Scale (K10)

The Kessler Psychological Distress Scale (K10) [49] is used as screener in this study. The K10 consists of 10 items measuring the amount of distress and severity of the psychological symptoms. Due to outstanding psychometric characteristics, the K10 is a worldwide used screener [49-51]. The 10 items are scored from 1 to 10 and a sum-score can range between 10 and 50. The cut-off score is 20. The K10 measures depression and since this study also includes anxiety, we use an extended version of the K10, developed by The Netherlands Study of Depression and Anxiety (NESDA, ). The extended version contains five extra items for anxiety. The anxiety items can only be answered "yes" or "no". Compared to the K10, the extended K10 has a higher sensitivity and specificity for detecting both depressive and anxiety disorders [52].

Respondents screen positive when they score higher than the cut-off score of 20 and/or reported at least one "yes" on the anxiety items. Respondents with a negative screener were excluded from the study.

##### Composite International Diagnostic Interview (CIDI)

The CIDI (version 2.1), a structured interview developed by the World Health Organisation [33], enables trained interviewers to assess psychiatric diagnosis defined in the Diagnostic and Statistical Manual of the American Psychiatric Association, 4^th ^edition [53]. The assessment typically lasts 30–75 minutes, depending on the mental state of the respondents [54]. In this study, current mental statuses within the last two months will be considered.

#### Primary outcome

##### Depressive symptoms

We will use the Inventory of Depressive Symptomatology (IDS) to measure depressive symptoms. The IDS consists of 26 items and the total score varies between 0 and 79. Scores below 14 indicate absence of depressive symptoms. We use this cut-off score as an indication of recovery from depressive symptoms [44,46]. Internal consistency is high for the IDS with Cronbach's alpha being .92 [44].

##### Anxiety symptoms

For identifying anxiety disorders we will use the Hospital Anxiety and Depression Scale (HADS) [31], which is usually used to identify anxiety disorders among patients in non psychiatric settings. The HADS consists of 7 items. Item responses are on a 0 to 4 scale (0="none" and higher ratings reflect greater degrees of symptom severity). Total-scores range from 0 to 21. The HADS showed good homogeneity and reliability, with Cronbach's alpha ranging from .81 to .84 in different normal and clinical Dutch samples [55].

##### Dysfunctioning

We will measure daily functioning of the patient via four questions of the Work and Social Adjustment Scale (WSAS) [43,56]. The patient gives an estimate, on a scale from 1 to 10, of the perceived dysfunctioning in daily live. The questions contain four domains: household, work, social relations and social activities.

#### Secondary outcome

##### Quality of life

Quality of life is measured through the MOS Short-Form general health survey (SF-20) which identifies the health related quality of life [57]. This self-report questionnaire (20 items) consists of six scales covering mental health, perceived health (mental and physical), social and role functioning, physical functioning and pain. A high score on the SF-20 indicates a high level of functioning, except for the subscale physical pain where a higher score indicates a higher level of pain. The alpha of the scales varies between .80 and .91 [57,58]. Quality of life is furthermore assessed using the EuroQol Questionnaire (EQ5D) [59], which is a validated tool for measuring general health related quality of life. It consists of five items (mobility, self-care, usual activities, pain/discomfort and anxiety/depression), each of which is rated as causing 'no problems', 'some problems' or 'extreme problems'. The EQ5D distinguishes 486 unique health states. Each unique health state has a utility score which ranges from 0 (poor health) to 1 (perfect health). EuroQol also contains a Visual Analogue Scale (VAS) on which patients rate their own health between 0 (worst imaginable health state) and 100 (best imaginable health state). Recently, a study in The Netherlands measured and valuated the EQ-5D in a national setting, resulting in the 'Dutch EQ-5D tariff' [60]. This tariff is used to calculate utilities for EQ-5D health states for cost-utility analyses of Dutch health care programmes and treatments.

##### Symptoms

The Four Dimensional Symptom Questionnaire (4DSQ) is developed for GPs to distinguish depression, anxiety and somatisation from more general distress [61]. The distress scale exists of 16 items and scores range from 0 – 32, the depression scale exists of 6 items and scores range from 0 – 12, the anxiety scale exists of 12 items and scores range from 0 – 24 and the somatisation scale exists of 16 items where scores range from 0 – 31. A higher score on the symptomatology indicates more serious complaints. The 4DSQ is a valid self-report questionnaire [61].

##### Alcohol use

The Alcohol Use Disorder Identification Test (AUDIT) [32] is a list of 10 questions about alcohol use. This test measures three different domains: quantity and frequency of alcohol use, the symptoms of alcohol dependency and the negative reactions on/consequences of alcohol use [62]. The score ranges from 0 – 40. A score of 9 or higher indicates a risk of an alcohol problem and a score of 13 and higher indicates the presence of an alcohol problem [63]. A study of Maisto et al. [62] shows that the AUDIT is a reliable and valid instrument. A score of 20 or higher on the AUDIT means exclusion from this study. Indices of internal consistency, including Cronbach's alpha and item total correlations, are generally in the .80's [64].

##### Personality

The Neuroticism, Extraversion, Openness to New Experience-Five Factor Inventory (NEO-FFI) is a questionnaire that contains 60 items on the five personality domains; neuroticism, extraversion, openness to experience, agreeableness and conscientiousness. The item responses are given on a 0 – 5 scale (strongly disagree, disagree, neutral, agree, strongly agree) [65]. A study of Weinstock and Whisman [66] shows that neuroticism is commonly manifested together with depression and anxiety disorders, therefore we only use those 12 items that identify neuroticism. Internal consistency ranges from .68 to .86 [67].

##### Health care utilisation

We use the Trimbos and iMTA questionnaire on Costs associated with Psychiatric illness (TiC-P) [68] to collect data on direct and indirect costs from the patients. The first part of the TiC-P measures the amount of medical care received by the participants, while the second part measures work productivity. We are going to confirm the actual received care, by checking the patients' care status and received care (e.g., medication, number of contacts in primary care and referral policy) during the stepped care project at the general practice settings and the specialized mental health care centers.

##### Health related absenteeism

For the measurement of health related absenteeism, we used the earlier mentioned TiC-P.

##### Costs

We will examine the societal costs which are based on the health care utilisation and health related absenteeism.

##### Satisfaction with care

This is measured via the QUOTE, which stands for Quality Of care Through the patient's Eyes. This questionnaire gives insight in the patient's opinions and believes about the quality of the received care [69]. There are different questionnaires for different diseases. For our study we use the questionnaire for anxiety and depression developed by NIVEL. In the first part of the QUOTE the perceived importance of care-aspects in anxiety and depression are measured. This part contains of 18 items and item-responses are on a 0 – 4 scale (not important – extremely important). The second part of the QUOTE consists of 18 items on the patients experience with the last received care. Item-responses on the second part are on a 0 – 5 scale (yes – no: or no experience).

##### Mastery

We assess the amount of perceived control through the Pearlin Mastery Scale [70]. This scale has 7 items measuring how much an individual perceives having control over things in his or her life. Items are rated on a 4-point scale with higher scores indicating more perceived control. Outcome-scores range from 7 – 35. The questionnaire has good psychometric properties [70] (Table 1).

**Table 1:** Overview of the selected instruments

			**Time of measurement**			
**Aim**	**Measurement**	**Screening**	**Baseline** **(pre-test)** **T0**	**T1**	**T2**	**T3**

						

Demographic variables	Sex	X	X	X	X	X

	Date of birth	X	X	X	X	X

	Native country	X				

	Education		X			

	Income		X			

	Marital status		X			

	Motivation		X (T)			

*Screening*						

Symptoms of depression and anxiety	Extended K10	X	X (T)			

						

*Primary Outcome*						

Symptoms of depression	IDS		X	X	X	X

						

Symptoms of anxiety	HADS-A		X	X	X	X

						

*Secondary Outcome*						

Diagnosis	CIDI		X (T)			X (T)

						

(Dys) Functioning	WSAS		X (T)	X	X	X

						

Quality of life	SF 20		X	X	X	X

						

Symptomatology	4DKL		X			X

						

Alcohol Use	AUDIT		X			

						

Perceived health	EuroQol		X	X	X	X

						

Neuroticism	NEO-FFI		X			

						

Costs of care	TIC-P		X	X	X	X

						

Quality of care	Quote					X

						

Mastery	Pearlin Mastery Scale		X			

						

Suicidal risk	MINI suicide		X (T)			

### Sample Size

A meta-analysis on the effects of psychological treatment on patients with sub-clinical depression shows an effect size of 0.40. Based on a power of 0.80 in a two-tailed test and an alpha of 0.05, we need 100 patients in each condition. Therefore, the total sample size is set at 200.

### Statistical Analysis

All analyses will be conducted according to the intention-to-treat principle. All respondents who have been randomised will be included in the analyses examining the effects of the intervention. Missing data will be imputed using regression imputation. The effectiveness of the stepped care program is measured via the primary outcomes: the HADS (anxiety) and IDS (depression). It is useful to know not only wether the effectiveness is statistically significant, We will use Cohens' *d *[71] to measure the size of the effect. Furthermore, GEE-analysis will be used to examine differences in speed of recovery between the two groups [72]. Additionally, we will report the percentage of patients who have recovered at the moment of the last assessment. In order to do so we will calculate the relative risk.

## Discussion

This paper describes the design of a study to investigate the effectiveness of stepped care for patients with minor and/or major depressive and anxiety disorders in comparison with care as usual in general practice. The following four steps are included in the stepped care model: watchful waiting, guided self-help, PST and pharmacotherapy and/or referral to mental health care. All these interventions are evidence based but there is a lack of studies regarding the effectiveness of stepped care as a whole. Results of this study could offer encouragement for the implementation of an effective stepped care model.

A major strength of this study is that it is a pragmatic randomised trial. In a pragmatic trial, patients and therapists are the same as those seen in daily practice. This means that the sample of patients may be quite heterogeneous (may have mild to severe depression/anxiety with or without psychiatric or somatic co-morbidity) and that the therapists (psychiatric nurses or psychologists) have average qualifications (instead of top level therapists from an academic centre). This enhances external validity which means that the results of this study will reflect the 'real' effects of daily practice. If an intervention is shown to have a significant beneficial effect in a pragmatic trial then it has been shown not only that it can work, but also does work in real life [73].

This advantage of the study has also some risks. First of all, it is difficult to maintain treatment integrity when conducting a study in day-to-day clinical practice and using personell not specifically hired for research. We hope to minimize this limitation by organizing strict supervision of the care managers.

Secondly, it is possible that there is a bias in our GP recruitment. To conduct this study, we need GPs who work together with a psychiatric nurse. This could mean that these particular GPs are aimed at mental health problems more than their colleagues who work without psychiatric nurse in their practice. If the GPs in our study pay more attention to mental health problems then GPs without psychiatric nurses, then our care as usual might be more adequate then care as usual in general.

Another limitation of our study could be the fact that we choose to recruit people with a diagnosis who do not receive any form of care and/or medication. There is a possibility that these patients have visited the GP before, but were not recognized as mental health patients. These patients might differ in a number of aspects from those who were recognized as mental health patients by their GP. It is possible that this leads to selection bias. To examine this we will conduct a non-response research.

It is widely acknowledged that depression recognition and management in primary care can be improved. As a consequence many collaborative care models, or disease management strategies, have been developed in recent years. The core elements of these care models are (1) an enhanced case management role for nonmedical specialists such as practice nurses and (2) integrated working relationships between primary care and specialist/secondary services [74]. In general these models seem to improve depression treatment [75]. Previous research into a partly implemented stepped care model in secondary services has shown cost-effectiveness compared to care as usual [29,76]. However, to our knowledge, our trial is the first trial with a fully implemented stepped care model for depression and anxiety treatment in primary care compared to care as usual.

## Competing interests

The authors declare that they have no competing interests.

## Authors' contributions

AvS and PC obtained funding for the study. WS coordinates the recruitment and data collection during the study. AB and PC are responsible for the overall supervision. HvM, AvS and PC provided the setting of the project. All authors provided comments, read and approved the final manuscript.

## Pre-publication history

The pre-publication history for this paper can be accessed here:


